# Dental Hints Department

**Published:** 1906-07

**Authors:** B. H. Teague

**Affiliations:** Aiken, S. C.


### DENTAL HINTS DEPARTMENT

Conducted by B. H. Teague, D. D. S., Aiken, S. C., to whom all contributions to this department should be sent.

Abscessed Tooth Caused Lockjaw.—A farmer living near Houston, Tex., died from lockjaw, caused by an abscessed tooth.—*Summary.*

Two Trays From One.—Take an ordinary flat-bottom tray and with a hack-saw cut midway through the handle. This makes two good partials at the expense of one.—*Dental Brief.*

Malocclusion.—The careful observer may detect irregularities in the positions of the temporary teeth that are sure indications of malpositions among the permanent teeth *that are not yet erupted.—E. A. Bogue, International Dental Journal.*

Local Anesthetic.—The following is an admirable local anesthetic to be used before the opening of boils, felons, etc.: Chloroform, ten parts; ether, fifteen parts; menthol, one part. Spray on part with an atomizer.—*Monthly Cyclopedia of Pract. Medicine.*

Mummifying Root Canal Filling.—A mummifying root canal filling that has been recommended consists of a liquid composed of equal parts of formaldehyde and creosote, and a powder consisting of 5 parts of thymol, 12 parts of dry alum and 40 parts of zinc oxide. If liquid should be cloudy a few drops of alcohol will clear it.—*Stomatologist.*

Pruritis.

Acid, carbolici, min. xx.

Cocainæ hydrochlorat., gr. x.

Vaselin,

Fiat ung.—*Dr. M. Morris, So. Med. & Sur.*

Pulp Devitalization.—I induce anesthesia by the use of cocain and suprarenalin, mixing and using any dilutant I choose, as water, chloroform, or oil of cloves. I have not yet found a pulp in the six interior teeth that will not anesthetize with but little pressure, without the use of rubber.—*T. E. Purcell, Western Dental Journal.*

Orthodontia and Rhinology.—Orthodontia is becoming recognized as a necessity in nose and throat treatment. Instead of the removal of turbinated bones to give room in the nasal tract, the better way is to expand the dental arch, which will normally enlarge the nasal opening and promote better breathing by purely natural means.—*Western Dental Journal.*

Died From Effects of Chloroform.— Mrs. Anton Williamson died in the chair of a dentist in Osage, Ia., April 4, after chloroform had been administered for extraction of teeth. One tooth had been extracted when the heart ceased beating. Upon reviving she complained of pain, and again losing consciousness, died before a physician could reach her.—*Summary.*

Bake, Not Broil, Porcelains.—Dr. J. E. Nyman says: "I am quite confident that we have all been fusing our porcelains too quickly. You can obtain the same result by prolonging the time of the exposure, and cutting down the intensity. We have been 'broiling' our porcelain instead of 'baking' it."—*Dental Office and Lab.*

Final Polishing of Plates.—In giving the final polish to rubber plates, I find that a polishing wheel made of about twenty-five thicknesses of unbleached muslin gives a better finish than chamois skin or a soft brush wheel. The edges of the circles of cloth become frayed and do not scratch. I can get a finer polish with this kind of polishing wheel than with anything else I know of.—A. C. Willman, *Dental Review.*

Polish for Porcelain.—Proceed as follows: Make a very soft paste of a saturated solution of spirits turpentine, gum camphor and pumice flour. Keep your polishing wheel wet with this mixture while repolishing the tooth. You will be pleased with the result.—Dr. W. H. Spaulding.—*Summary.*

Gold Inlays.—Gold inlays are a most excellent substitute for gold crowns in molars and bicuspids where the teeth are very badly broken down, and it is inevitable that their introduction and use in the hands of conscientious and skillful operators will prove a boon to mankind and a stimulus to the cosmetic in dentistry.—*Items of Interest.*

Dish for Acid Bottles.—Set your laboratory acid bottles in a glass dish or granite or other glazed tray, first covering the bottom with a thin layer of washing soda. The last-mentioned article takes up the drip from the neck of the bottles, also to some degree the heavy fumes. If it is desired to have a dish to each bottle, the sponge dishes in use by bank cashiers and dentists generally when counting their money, answer the purpose perfectly.—*Dental Office and Lab.*

LOBELIN.—In spasmodic retention of urine due to stricture, give gr. 1/134, every ten minutes, after atropin gr. 1/250, in a little hot water. Dissolve gr. 1/67 lobelin in twenty drops of hot water and inject into the deep urethra.—*Candler. Amer. Jour. of Clen. Med.*

USE FOR SLATE PENCIL.—The suggestions that I give are not all original with me, and in calling them helpful I mean they have been so to me. One is: A common slate pencil is better for teasing solder than an instrument. The solder does not adhere to it and it does not heat up and burn the fingers. It can also be used for stirring fusible alloy.—*A. C. Willman, Dental Review.*

TO KEEP YOUR FLASKS CLEAN.—Place a small quantity of sodium bicarbonate in the vulcanizer before using and you will be surprised how clean you can keep your flasks. They are readily and easily cleaned after such use. A pair of flask tongs will aid in keeping the hands clean, and one can handle the hot flasks to better advantage.—*H. E. Davis, Review.*

SODIUM PERBORATE.—The preparations of $H_2O_2$ usually employed possess the disadvantage of being acid in reaction. Sodium perborate offers advantages of the highest order. It is of alkaline reaction and perfectly stable and can be preserved indefinitely even when exposed to the air or kept in unstoppered bottles. One kilogram contains 104 grains of active oxygen which becomes available on mixing with distilled water. It may be used in solution or in powdered form, as when in contact with the tissues, inflamed or normal, a sufficient amount of humidity is always present to cause the evolution of oxygen.—*L'Odontologie.*

FILLING CHILDREN'S TEETH:—I find that it is better to postpone attempting permanent operations even when small cavities are to be dealt with in children's teeth. I find that with the erupting first molars it is very much better not to attempt to make gold fillings, but to wait until three or four years later.—*J. W. Wassall, Review.*

A WORD OF CAUTION:—Never fill a root canal at the same sitting after the removal of a pulp with cocain, particularly when adrenalin is combined with the solution. First, because of the secondary hemorrhage and, second, the tissue surrounding the apical space being anaestheticized renders it impossible to work with the same degree of accuracy as when this space is sensitive, especially when forcing in the root filling.—*S. Marshall Weaver, Cleveland, O.*

To Remove a Morbid Growth of Gum Tissue from a Cavity:—Frequently we have cases when a morbid growth of gum tissue fills, or partially fills, a cavity. Its removal is not only somewhat painful, but it is also a mean piece of work to cut it away on account of the excessive hemorrage. In the majority of such cases it can be removed neatly and with dispatch, painlessly and bloodlessly, by ligating with a piece of silk to either the tooth with the cavity or the adjoining one, whichever is the most convenient. This cuts off the blood supply and reduces to a minimum the pain when cutting away the growth with a side motion of a small flat burnisher or other suitable instrument. If a bad case, touch with trichloracetic acid. For those who have not tried this method of removing this troublesome growth the result will be surprising.—*Dental Owce and Lab.*

Alphozone (cooh. $ch_2$. co)$_2$o$_2$.—Frederick Sterns & Co. is a splendid article for bleaching teeth, that is, where the pulp has been removed and the tooth is discolored. I wished to bleach a tooth. I placed a quantity in the cavity, moistened it and sealed it up for two days. The result was better than I expected so I again applied it for the same period of time when the tooth was perfectly bleached to correspond with the other teeth adjoining.—*Dr. J. P. Reardon, Lawrence, Mass.*

Uniting a Broken Plate.—It is often difficult to securely wax together a broken plate, especially a lower partial. The attempt to hold the broken pieces in one hand while you attach them with the other makes you wish you had three or four hands. If a strip of wax, such as the dental depots use to attach a set of teeth to, be softened and laid on a flat, unyielding surface, a piece of glass for instance, the broken plate may be pressed, teeth down, into the wax, using both hands to hold the pieces in proper position. When you let go, they will remain in place and you are free to examine carefully and wax together.—*A. C. Wellman, Dental Review.*

The Reason for "Patents":—And here it may be remarked that there is a reason for the "patent-medicine fake."

Many patent and "proprietary" compounds are distinctly useful. Sometimes they fill a void in what we are wont to term "legitimate" therapeutics. Most of those that are worthy of confidence have been manufactured in the first instance, from the prescriptions of physicians. Many patent compounds have become distinct evils, whatever they may been originally. —*Amer. Jour. Clin. Med.*

Trimming and Finishing a Lower *Plate.*—More than one lower denture has been laid upon the upper shelf of the pantry, or has been deposited within the depths of an old-fashioned sugar bowl, to await the resurrection of the just, because the plate was not properly finished. Most of the time the flanges, or rims, are left too long. They ride upon the tissues of the part of the mouth with which they come in contact, like a buoy at sea with the tide. In talking, the tongue displaces a plate like this, and in the act of mastication the buccinator muscles lift it up, and soon the movement, and the pressure upon it, cause the rims to cut their way into the soft parts, and an almost unbearable abraison or sore results, so that the plate is removed and laid aside, in many cases never to be tried again, because "it don't fit." Some patients will endure this kind of annoyance for a while, and then return for relief, but most of them condemn the work and the worker in unmitigated terms, and refuse to be further tortured. All this can be avoided by trimming the plate, before dismissing the patient, so that the edges do not bear on the soft parts, and by having it rest squarely upon the ridge without impinging upon the muscles at the root of the tongue, or upon the attachments upon either buccal side. In many cases the muscular tissues and soft parts are raised when the tongue is raised, and extend above the ridge; this condition must be well looked to, so that the plate may be adjusted by proper trimming until it is not lifted out of place when in use. All the edges of the plate should be well rounded and smooth. On the buccal and lingual surfaces, concavities for the sides of the tongue and the folds of the cheek are found to materially assist in retention, and add to the comfort of the patient.—*J. A. Hartman, Dental Office and Laboratory.*

The Teeth in Sickness.—I have seen many mouths ruined by neglect during fevers. A dentist often never hears that his patient has had fever till the latter comes to have a lot of dental wrecks treated. I think it is the bounden duty of the doctor to enforce dental hygiene in all fevers and prolong cases of iliness, when patients are beyond the sphere of their dentist.—*F. J. Turnbull, Dental Record.*

Gold Solders.—Formerly gold solders were stamped with the karat mark. Eighteen-karat solder meant solder containing eighteen parts of pure gold to six of alloy, and eighteen-karat gold plate meant the same, the difference being in the character of the alloying metals, the solder containing some lower fusing metal that lowered the fusing point of the mass. Still, both being of the same karat, one was as fine as the other. —*Dr. S. H. Guilford, Stomatologist.*

Chloretone.—A ten or twenty per cent solution of chloretone in 75 per cent alcohol is valuable as a topical application previous to the use of the hypodermic needle in the gums. The alcohol cuts the mucus and leaves the membrane absolutely clean, and sterilization of the field of operation results, while the anesthetic action of the chloretone is had in the tissues and the needle can be used with the minimum of pain and no possiblity of septic infection.—*T. A. Gormley, Dental Register.*

Silver Nitrate with Cement Fillings.—A cement filling, zinc oxyprosphate, placed upon a surface treated with silver nitrate, will, for some reason, last a great deal longer and will be a great deal better mass than the same mass not having the peculiar effect it gets from this film of silver albuminate. A great many, no doubt, have seen the effect of silver nitrate upon a surface which has been infected to a very slight depth. That in time will become a polished black surface and further decay will never result. Now, if in deeper cavities we can make partial preparation and apply silver nitrate, and have it last longer than otherwise, it is worth knowing.—*W. V-B. Ames, Dental Review.*

Stanno-percha.—The new and excellent properties of stanno-percha very materially increase its usefulness. Gutta-percha and Hill's stopping have been used for a long time for the capping of pulps and as an insulator for large metal fillings. However, Fletcher's artificial dentin, asbestos felt and other materials are now used largely. In the future, we believe, stanno-percha will be used exclusively for such purposes, because it is so easy, and without difficulty, to place it in correct position. Amalgam will readily unite with the tin present, and either sponge gold or gold foil may be readily worked into the slightly softened layer. As a temporary filling material or for retaining arsenical dressings, stanno-percha will be of much value and perferred over all other materials. Every dentist may easily prove to himself Cruschtschoff's or my own assertions, and he will find in stanno-percha a labor and time saving filling material of great value.—*Dr. A. Scheme,Eva.*

Is the Painless Dental Operation Profitable.—From the patient's standpoint painless operations may mean larger fees to pay, but not in such porportion as to make them less desirable. From the patient's standpoint they may contribute to good health, more comfort, more nerve strength, more happiness and more durable work.

From the dentist's standpoint it will be so profitable in every way that almost without exception he would leave the practice of dentistry rather than return to his former more painful methods. If to him *profitable* means more patients it certainly will bring them, for everywhere everybody, himself included, is looking and planning to avoid pain, the greatest menace to happiness. If *profitable* means more reward in fees for his services (a very legitimate ambition for worthy service) he certainly will get it, for none pay so cheerfully and liberally as appreciative patients, and most people would gradly pay more not to be hurt than they would for the operation itself. If *profitable* means to him a consciousness of having done for humanity a great good—a worthy reward for a worthy man—he will have a boundless joy in his professional life which the narrow, selfish operator never has dreamed possible. From the dentist's standpoint it is certainly profitable, no matter from what way he views it, and will bring him returns in many times larger proportion for the outlay than any other special efforts he can make.—*Dr. W. A. Price, Den. Mag.*

Cleaning Teeth by the Dentist.—The time has come when the dental profession should act, and act wisely, in regard to educating the public on the subject of cleanliness of the oral cavity. Mastication is a first step in a series of processes for the digestion and assimilation of food and unless this process is thoroughly and hygenically done the digestive system and the general health must suffer the consequences. Food can not be well masticated with diseased and aching teeth, nor is the food clean and healthful after being masticated by such teeth covered with decomposed vegetable and animal matter, which not only vitiates the food before it reaches the stomach, but poisons the life giving oxygen before it reaches the lungs. The perfect cleansing of infected gums and teeth is now recognized as one of the most difficult operations that the dentist has to perform, and many a dentist when cleaning a set of teeth covered with tartar has wished that he could take those teeth from the mouth, scrape them, boil them in some antiseptic, polish them on the lathe and place them back in their original sockets, but as this is not feasible he, through three or four sittings, scrapes and stearilizes almost as well as he could do in a few moments if the teeth were out of the mouth and in his hands.—*Dental Review.*

LOCAL ANESTHETIC.—R. Cocainæ hydrochoratic, gr. vi.
Acidi carbolici, m. ii.
Aq. menth. piperitæ, q. s. ad. f. oz.i.

M. Sig.—Use as a local anæsthetic hypodermically.

Now, if you feel like paying $1.50 for a local anæsthetic, the constituents of which you don't know, or if you feel like paying fabulous prices for putrescent pulp mixtures, the constituents of which you do not know, you may do so; but if you don't feel justified in doing that, you can use this local anæsthetic formula which I suggested, which is a modification of other formulas I have seen. My vehicle is peppermint water, because it contains enough methol to mask the bitter taste of cocaine. Most of these local anæsthetics are bitter. Peppermint water is not strong enough, however, to keep fungus growths from forming in this solution, so we can add a disinfestant, carbolic acid, for the purpose of keeping sterile our solution. I have prescribed only one ounce, and if I have freshly prepared peppermint water the acid used, this solution will keep sterile for the length of time it takes me to use one ounce of the fluid.—*Dr. Buckley, Dental Review.*

"EAREACHE" AND THE TEETH.—A physician near here treated a patient for three weeks for earache and neuralgia but failed to relieve her, except by the use of sedative. Finally she complained that a lower wisdom tooth felt longer than the rest of her teeth. Her physician came to the conclusion that probably that tooth was the cause of her suffering. I was called in to extract it. She was pillowed and propped up in a Morris chair. Constant pain soon wears down the strongest constitutions and this woman was certainly in a debilitated condition. She also had a heart affection which did not help matters. Her physician and several of the neighboring women were present. I never wish my competitors bad luck but I certainly did wish one of them had this case. I got out my "torture goods" and proceeded to remove that tooth in the latest and most scientific manner. I succeeded most beautifully—that is I broke the tooth the first attempt. After assur ing her that the breaking of the tooth would give her as much relief as if I had extracted it mustered up enough courage to take a look at the remains. To my surprise I discovered I could remove it quite easily. When I got that tooth out she heaved a sigh of relief, for the pain ceased almost instantly. In a week she could do her own work.—*J. A. McPhail, American Journal of Clinical Medicine.*

